# Comparison of small-bowel colon capsule endoscopy system to conventional colonoscopy for the evaluation of ulcerative colitis activity

**DOI:** 10.1055/a-0982-2786

**Published:** 2019-10-01

**Authors:** Samuel N. Adler, Yago González Lama, Virginia Matallana Royo, Cristina Suárez Ferrer, Avraham Schwartz, Ariella Bar-Gil Shitrit

**Affiliations:** 1 Digestive Disease Institute, Shaare Zedek Medical Center, Jerusalem, Israel; 2 Gastroenterology and Hepatology Department, Puerta de Hierro University Hospital, Majadahonda, Madrid, Spain

## Abstract

**Background and aims** Diagnosis and monitoring of ulcerative colitis (UC) includes conventional colonoscopy. This procedure is invasive and does not exclude small-bowel Crohn’s disease (CD). Current therapeutic goals include mucosal healing which may lead to an increased number of endoscopic procedures in many patients. The small-bowel colon capsule endoscopy (SBC-CE) system visualizes the small bowel and colon. The aim of this study was to evaluate the performance and adverse events of SBC-CE in patients with UC.

**Methods** This was a prospective, feasibility study involving two study sites. Patients with active UC underwent SBC-CE and colonoscopy. Kappa statistics were performed to assess the agreement between SBC-CE and colonoscopy. Adverse events (AEs) data were collected throughout and following the procedure.

**Results** In total, 30 consecutive patients were recruited, and 23 of those were included in the final analysis. For the primary end point, evaluation of the extent of UC disease in the colon, the percent agreement between SBC-CE and colonoscopy was moderate (56.5 %); kappa coefficient 0.42. The percent agreement between SBC-CE and colonoscopy for UC disease activity, based on Mayo endoscopic sub-score, was 95.7 %; kappa coefficient 0.86. Disease activity in the more proximal small bowel was detected in two patients with SBC-CE. No SBC-CE device-related AEs were reported.

**Conclusions** When comparing SBC-CE to conventional colonoscopy, there was a moderate agreement for the extent of UC disease and a very good overall agreement between the two modalities for UC disease activity.

## Introduction

Ulcerative colitis (UC) is a chronic inflammatory bowel disease (IBD) located in, but limited to, the colon. The inflammatory process involves the mucosa and submucosa and usually spreads proximally from the rectum to variable extents in the colon. The main manifestation of UC is diarrhea and rectal bleeding 1 2 . Clinicians have treated UC historically with the intent to achieve clinical improvement (clinical response) or even a symptom-free situation, which is known as clinical remission (CR). These clinical benchmarks determine the success of different treatments in both clinical practice and trials. However, researchers have reported a lack of correlation between clinical assessment and endoscopic healing 2 3 4 .

CR may be present in the absence of mucosal healing (MH), and the mere absence of symptoms is no longer a reliable treatment goal. Several publications have suggested that CR may be insufficient as a desired treatment target 5 6 7 8 . A high quality meta-analysis has suggested that UC patients successfully treated with documented MH will have improved outcomes that include a higher rate of long-term CR and a higher chance remaining free of a colectomy 9 . MH remained a prognostic factor regardless of whether a biologic or another therapy was used. In fact, a consensus of leading experts in IBD on selecting therapeutic targets recommends endoscopic assessment 3 to 6 months after initiation of UC therapy in symptomatic patients 10 and the U.S. Food and Drug Administration now mandates clinical improvement (patient reported outcomes) and MH for approval of new IBD therapies 11 12 .

Sigmoidoscopy or colonoscopy diagnosed MH is defined as the resolution of ulcerations and friability 13 14 and there are clinical circumstances that require endoscopic confirmation of the presence or absence of mucosal inflammation 15 . However, colonoscopy is invasive, expensive, and demands the use of endoscopic facilities, all of which may reduce the practicality of using endoscopy in the clinical setting. The small-bowel colon capsule endoscopy (SBC-CE) system is a non-invasive panenteric system that visualizes the mucosa of the small bowel and colon and may therefore monitor inflammatory activity and document mucosal healing. Furthermore, SBC-CE visualizes the entire gastrointestinal tract and may detect unsuspected small-bowel Crohn’s disease (CD) in patients treated for UC. Capsule endoscopy (CE) has been widely used in CD for small intestine evaluation; moreover, SBC-CE has recently been approved for panenteric endoscopy in CD and received a CE mark in 2016 and FDA clearance in 2017, but its usefulness in UC has never been evaluated. There have been previous attempts to evaluate the usefulness of CE in colonic diseases such as UC 16 17 18 19 , but data were insufficient to recommend colon capsule studies in the evaluation of IBD, probably because appropriate technologic development was still lacking 20 21 . The aim of this study was to evaluate the adverse events of a new SBC-CE platform in patients with known UC, to compare capsule endoscopic accuracy in assessing disease extent, and to compare capsule endoscopic accuracy in assessing disease activity in UC to that of conventional colonoscopy.

## Materials and methods

### Study design

This was a prospective, comparative, feasibility study. Patients were enrolled consecutively from two study sites, one in Israel and one in Spain, from June 2014 through February 2015.

### Ethical considerations

The study was conducted according to Good Clinical Practice guidelines and the Declaration of Helsinki, and experimental protocols met United States FDA guidelines and were approved by the investigative site’s institutional review committee (approved on March 2014 in Israel and June 2014 in Spain). This trial was registered at ClinicalTrials.gov, NCT02025777. Informed consent was obtained for all patients.

### Study participants

Eligibility to participate in the study was based on the inclusion and exclusion criteria ( Table 1 ). Inclusion criteria were: patients 18 years of age or older, had established UC, and patients had to have active disease with signs of fresh bleeding and/or bloody diarrhea (i. e. anemia based on complete blood count [CBC], specifically hemoglobin [HgB]) and/or at least one positive inflammatory marker (erythrocyte sedimentation rate [ESR], C-reactive protein [CRP]), within the past 3 months. Exclusion criteria included evidence of symptomatic stricture or other obstruction that would prevent capsule passage (see Table 1 for a complete listing of exclusion criteria).

**Table 1:** Subject inclusion and exclusion criteria.

Inclusion criteria	Exclusion criteria
Patient aged ≥ 18 years	Patient has Crohn’s disease
Patient has known UC according to physician discretion	Patient has antibiotic associated colitis
Patient has symptoms of fresh bleeding and/or bloody diarrhea and/or at least one positive inflammatory marker within the past 3 months from the following:	Stool positive for ova and parasites and for *Clostridium difficile* toxin within 3 months of enrollment
ESR	Other known infectious causes of increased symptoms
CRP	Known intestinal obstruction or current obstructive symptoms, such as severe abdominal pain with accompanying nausea or vomiting
CBC	Definite tight or long stricture seen on radiological exam
Patient indicated and eligible for a standard of care colonoscopy examination for evaluation of disease activity and not for routine screening for dysplasia or colorectal cancer	Non-steroidal anti-inflammatory drugs including aspirin (twice weekly or more) during the 4 weeks preceding enrollment
	Suspected gastrointestinal stricture, followed by patency capsule study or other imaging study that could not prove patency of the gastrointestinal tract
	Patient has had prior abdominal surgery of the gastrointestinal tract in the last 6 months, other than uncomplicated procedures that would be unlikely to lead to bowel obstruction based on the clinical judgment of the investigator
	Patient is expected to undergo MRI examination within 7 days after ingestion of the capsule
	Patient with a known gastrointestinal motility disorder
	Patient with known or suspected delayed gastric emptying
	Patient suffers from any condition, such as swallowing problems, which precludes compliance with study and/or device instructions
	Patient has Type 1 or Type II diabetes
	Patient has any allergy or other known contraindication to the medications used in the study
	Women who are either pregnant or nursing at the time of screening, or who are of child-bearing potential and do not practice medically acceptable methods of contraception
	Concurrent participation in another clinical trial using any investigational drug or device
	Patient suffers from a life threatening condition
	Patients with history or clinical evidence of renal disease and/or previous clinically significant laboratory abnormalities of renal function parameters

ESR, erythrocyte sedimentation rate; CRP, C-reactive protein; CBC, complete blood count; MRI, magnetic resonance imaging; UC, ulcerative colitis.

### Subject demographics

**Fig. 1:**
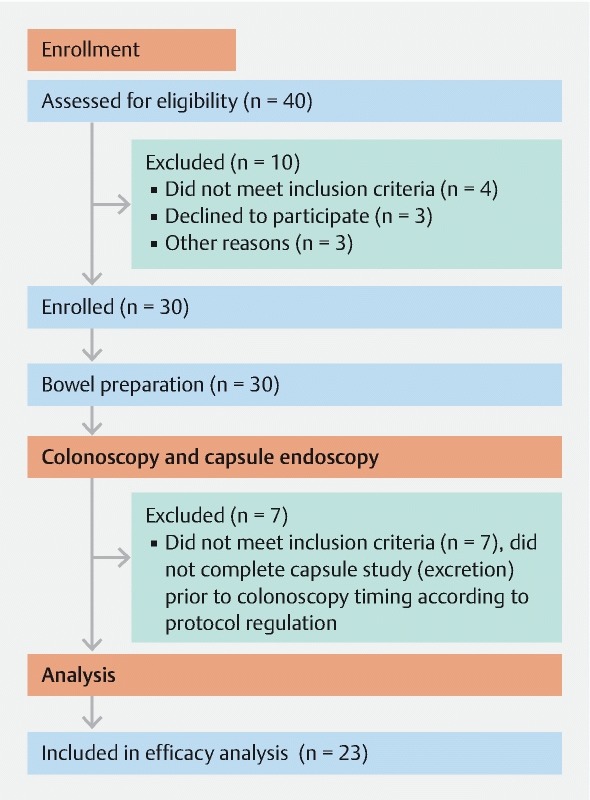
Study flow diagram for patient evaluation.

A total of 40 patients were assessed for eligibility and 30 consecutive patients were enrolled in the study, with a mean age of 45.4 years, of whom 53 % were male (n = 16) ( Table 2 ). Ten interviewed patients were not enrolled in the study. Four patients at the time of enrollment did not have active UC. Three patients at the time of the study were not available and three other patients withdrew their consent. Eighteen patients were enrolled in Israel and 12 in Spain. Of the 30 enrolled patients, 23 were included in the final efficacy analysis and completed both SBC-CE and colonoscopy procedures ( Fig. 1 ). Seven patients were excluded from the efficacy analysis due to protocol regulations that demanded same-day colonoscopy 10 hours after capsule ingestion. These seven patients underwent colonoscopy before capsule excretion and for that reason were excluded. The capsules were retrieved by colonoscopy in all seven patients. All excluded patients were followed-up and no complications were noted. A summary of patient demographics and baseline data is presented in Table 2 . None of the enrolled patients were taking NSAIDS.

**Table 2:** Subject demographics, baseline characteristics, and reason for referral for all enrolled patients and those included in the efficacy analysis.

Parameter	Enrolled (n = 30)	Efficacy analysis (n = 23)
Age at consent, mean ± SD, years	45.4 ± 13.5	43.6 ± 13.5
Gender, n		
Male	16 (53.3 %)	15 (65.2 %)
Female	14 (46.7 %)	8 (34.8 %)
Weight, mean ± SD, kg	70.0 ± 14.3	71.9 ± 14.5
Height, mean ± SD, cm	169.2 ± 9.0	170.2 ± 9.0
BMI, kg/m ^2^	24.3 ± 3.8	24.7 ± 3.9
Reason for referral		
Ulcerative colitis	30 (100.0 %)	23 (100.0 %)
CRP, mg/L	15 (50.0 %)	10 (43.5 %)
CBC 1 , SI units	2 (6.7 %)	1 (4.3 %)
ESR, mm/h	2 (6.7 %)	1 (4.3 %)
Has seen blood in the past 3 months	1 (3.3 %)	1 (4.3 %)

BMI, body mass index; CRP, C-reactive protein; CBC, complete blood count; ESR, erythrocyte sedimentation rate.

1 Anemia associated with active ulcerative colitis, i. e. hemoglobin [HgB].

### Test device

The PillCam Crohn’s Platform (Medtronic, formerly Given Imaging, Yoqneam, Israel) includes: the two-headed video capsule (PillCam Crohn’s capsule), PillCam Recorder Set, sensor array and sensor belt, and Rapid Real-Time Software and Given Workstation. The two-headed video capsule was specifically designed for the visualization of IBD lesions in the small bowel and colon. It has a panoramic field of view of 172 degrees in each head, and a frame rate from 4 up to 35 frames per second (in areas of fast transit) that adapts to the speed of capsule transit through the bowel.

### Endoscopic equipment

The study site located in Spain used an Olympus colonoscope model 180 (Olympus Europa, Hamburg, Germany). The study site in Israel used a Fujinon colonoscope model 250 W (FujiFilm Co, Tokyo, Japan).

### Study procedure

Patients were enrolled into the study after providing informed consent. Screening exams were performed within 30 days before the SBC-CE and conventional colonoscopy procedures. Screening assessments included age, height, weight, gastrointestinal symptoms, general medical history, concomitant medications, previous gastrointestinal procedure, and urine pregnancy test (if applicable).

One day before and during the SBC-CE procedure, patients were instructed to perform a bowel preparation procedure and follow a detailed dietary regimen. All bowel preparation products were standard bowel cleansing products. All sites and all patients followed the same preparation procedure as outlined in Table 3 . After capsule ingestion and completion of the SBC-CE procedure, the raw data and SBC-CE video were downloaded from the PillCam Recorder Set to the PillCam Workstation.

**Table 3:** Capsule endoscopy bowel preparation.

Day	Time	Procedure
Day ( – 1)	Until 14:00	Low residue diet
	After 14:00	Clear liquid diet
	20:00 – 21:00	1 L polyethylene glycol electrolyte lavage solution
Examination day	07:00 – 09:00	2 L polyethylene glycol electrolyte lavage solution
	10:00	SBC capsule ingestion
	11:00	Optional: 10 mg of Metoclopramide (only if capsule is in stomach)
	Upon small-bowel detection	0.5 bottle (88 mL) of sodium sulfate, potassium sulfate, and magnesium sulfate solution diluted to 240 mL water followed by 0.5 L water over the next hour
	3 hours later	0.5 bottle (88 mL) of sodium sulfate, potassium sulfate, and magnesium sulfate solution diluted to 240 mL followed by 0.5 L water over the next hour
	2 hours later	Optional (if capsule is not excreted): 0.5 bottle (88 mL) of sodium sulfate, potassium sulfate, and magnesium sulfate solution diluted to 240 mL followed by 0.5 L water over the next hour

SBC, small-bowel colon.

Following completion of the SBC-CE procedure, patients underwent colonoscopy on the same day. If the capsule was not excreted within 10 hours and was seen during the colonoscopy, it was removed during the colonoscopy procedure at the physician’s discretion. Patients with capsules that remained in the stomach or small bowel during the entire procedure were excluded from the efficacy analysis. The colonoscopy exam was performed according to the accepted guidelines for colonoscopy and was recorded on a VCR or DVD.

Patients were followed up by phone, 5 – 9 days after the SBC-CE procedure, to assess their well-being. Adverse events (AEs) data were collected throughout the study.

### Capsule endoscopy and colonoscopy video evaluation

Colonoscopy was evaluated locally by a single endoscopist, while SBC-CE videos were read by a single investigator from the other site. The sponsor trained gastroenterologists with extensive endoscopy experience to grade their colonoscopy findings and their findings of SBC-CE videos according to the Mayo endoscopic sub-score. For both SBC-CE and colonoscopy videos, readers reported pathologies in the colon and terminal ileum (TI), and the colon overall cleansing level. Bowel preparation scores were graded on a scale of 1 (poor) to 4 (excellent) as previously described 22 23 24 . Readers of SBC-CE videos additionally assessed any abnormal findings in the proximal small bowel. The readers of SBC-CE videos were blinded in reference to the colonoscopy findings and vice versa.

### Outcomes measures

The primary end point was the agreement between SBC-CE and conventional colonoscopy in the extent of UC disease (classified as proctitis, left-sided colitis, pancolitis, or inactive colitis).

Secondary end points included agreements between SBC-CE and conventional colonoscopy in UC disease activity (classified as active [mild, moderate, severe] or remission [inactive]), based on the Mayo endoscopic sub-score. Adverse events were assessed.

### Statistical and data analysis

As this was a study to determine proof of concept of the use of the new SBC-CE platform instead of colonoscopy, no power calculations were made in the determination of sample size.

Summary statistics for demographic and other baseline characteristics were calculated for the total study population and patients included in the efficacy analysis. Patients were excluded from the efficacy analysis if they withdrew from the study, if the capsule remained in the stomach or small bowel during the entire procedure, if colonoscopy could not be performed, or if there was a technical failure in the system.

For evaluation of UC disease activity, the Mayo endoscopic sub-score was used. This classifies UC as mild (score 1: presence of erythema, decreased vascular pattern, mild friability), moderate (score 2: marked erythema, absent vascular pattern, friability, erosions), severe (score 3: spontaneous bleeding, ulceration), or non-active disease (score 0) 25 .

The extent of UC disease was classified as proctitis, left-sided colitis, pancolitis, or inactive colitis. A standard four-point grading scale system (excellent, good, fair, poor) was used to measure the colon overall cleansing level as detected by both modalities 26 .

The kappa statistic (κ) was calculated with 95 % confidence interval to evaluate the agreement between SBC-CE and colonoscopy for UC disease activity and extent, and the colon overall cleansing level. Values of kappa near zero indicated agreement no better than expected by chance, while values near 1 indicated perfect agreement. Kappa was judged as providing ‘very good’ agreement if 0.81 ≤ κ ≤ 1.0, ‘good’ agreement if 0.61 ≤ κ ≤0.80, ‘moderate’ agreement if 0.41 ≤ κ ≤ 0.60, ‘fair’ agreement if 0.21 ≤ κ ≤ 0.40, and ‘poor’ agreement if κ ≤ 0.2 27 .

Patients were excluded from AE analysis if they withdrew for any reason, apart from an AE. Adverse event analysis was assessed by characterizing each reported AE by type, severity, duration, and relationship to the study procedure/device.

## Results

The percent agreement between the SBC-CE system and conventional colonoscopy in the evaluation of the extent of UC disease was 56.5 % (95 %CI: 34.5 – 76.8 %) with kappa 0.42 (95 %CI: 0.16 – 0.68) ( Table 4 ). The percent agreement between the modalities for the extent of UC disease was 78.3 % (95 %CI 56.3 – 92.5 %) with a kappa coefficient of 0.61 (95 %CI 0.34 – 0.89) ( Table 5 ) when proctitis and left-sided colitis were categorized together.

**Table 4:** Correlation between SBC-CE and optical colonoscopy with regard to extent of ulcerative colitis disease per primary end point.

	Colonoscopy findings				
	Proctitis, n	Left-sided colitis, n	Pancolitis, n	Inactive colitis, n	Total, n
SBC-CE findings					
Proctitis, n	5	3	0	0	8
Left-sided colitis, n	2	2	0	1	5
Pancolitis, n	0	3	2	1	6
Inactive colitis, n	0	0	0	4	4
Total, n	7	8	2	6	23

SBC-CE, small-bowel colon capsule endoscopy. Percent agreement between SBC-CE and colonoscopy = 56.5 % (95 %CI 34.5 – 76.8 %), kappa coefficient = 0.42 (95 %CI 0.16 – 0.68).

**Table 5:** Correlation between SBC-CE and colonoscopy for extent of ulcerative colitis disease when left-sided colitis and proctitis are combined.

	Colonoscopy findings			
	Left-sided colitis/proctitis, n	Pancolitis, n	Inactive colitis, n	Total, n
SBC-CE findings				
Left-sided colitis/proctitis, n	12	0	1	13
Pancolitis, n	3	2	1	6
Inactive colitis, n	0	0	4	4
Total, n	15	2	6	23

SBC-CE, small-bowel colon capsule endoscopy. Percent agreement between SBC-CE and colonoscopy = 78.3 % (95 %CI 56.3 – 92.5 %), kappa coefficient = 0.61 (95 %CI 0.34 – 0.89).

The percent agreement between SBC-CE and colonoscopy for UC disease activity, based on the Mayo endoscopic sub-score, was 95.7 % (95 %CI 78.1 – 99.9 %) with a kappa coefficient of 0.86 (95 %CI 0.60 – 1.00) ( Table 6 ). Colonoscopy found five patients in remission (Mayo endoscopic sub-score 0). SBC-CE agreed that four of these five were in remission; the fifth patient was judged to have Mayo endoscopic sub-score 1.

**Table 6:** Correlation between SBC-CE and colonoscopy for ulcerative colitis disease activity, based on Mayo endoscopic sub-score.

SBC-CE findings	Colonoscopy findings		
	Active, n	Remission, n	Total, n
Active, n	18	1	19
Remission, n	0	4	4
Total, n	18	5	23

SBC-CE, small-bowel colon capsule endoscopy. Percent agreement between SBC-CE and colonoscopy = 95.7 % (95 %CI 78.1 – 99.9 %), kappa coefficient = 0.86 (95 %CI 0.60 – 1.00).

**Fig. 2:**
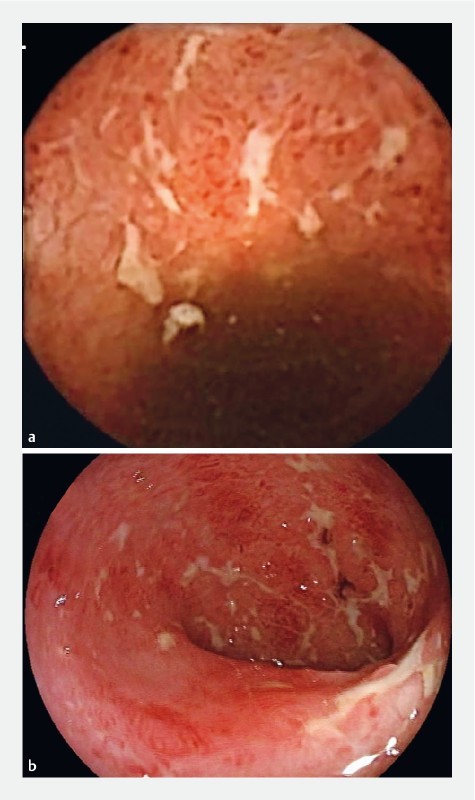
Images of stellate ulcers in the rectosigmoid as detected with SBC-CE ( **a** ) and colonoscopy ( **b** ).

**Fig. 3:**
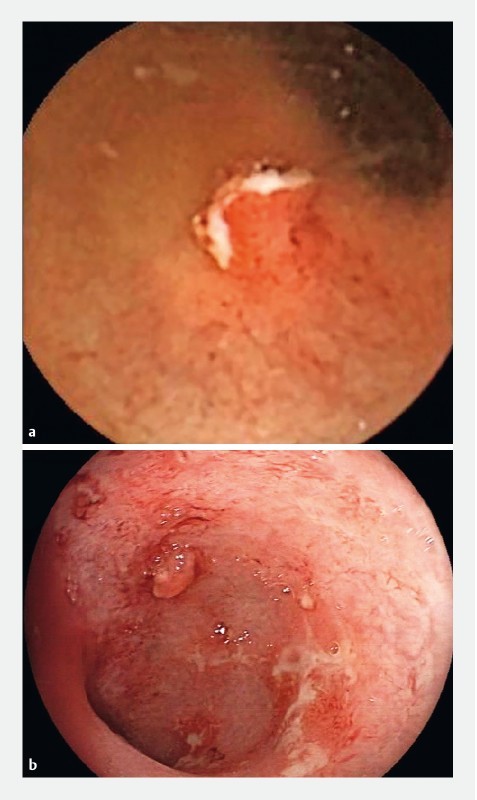
Images of ulcerative polyp in the rectum as detected with SBC-CE ( **a** ) and colonoscopy ( **b** ).

Images of findings detected with SBC-CE and colonoscopy are shown in Fig. 2 and Fig. 3 . Of the 23 patients included in the performance analysis, three patients (13.0 %) had findings in the small bowel. One patient had ulceration in the TI, as detected by SBC-CE. The finding in the TI was confirmed with colonoscopy. SBC-CE identified a second patient with ulceration and erythema in the TI and ulceration in the jejunum. In this case, no findings were detected in the TI with colonoscopy. The capsule reader changed the diagnosis from UC to CD in this patient. A third patient had ulceration detected in the proximal small bowel with SBC-CE.

For the colon overall cleansing level, as detected by both modalities (CE vs colonoscopy), the percent agreement was 91.3 % (95 %CI 72.0 – 98.9 %), with a kappa coefficient of 0.62 (95 %CI 0.16 – 1.0).

Of the 30 enrolled patients, three (10.0 %) had at least one procedure-related AE ( Table 7 ). Two patients (6.7 %) experienced nausea and vomiting, which were related to bowel preparation. One patient (3.3 %) experienced abdominal pain, which was related to colonoscopy. No patients had AEs related to the SBC-CE device, and no patients had any serious adverse events (SAE) related to the procedures.

**Table 7:** Summary of adverse events.

Variable	Number of patients (n = 30)
Patients with ≥ 1 procedure-related AE	3 (10.0 %)
Bowel preparation-related 1	2 (6.7 %)
Colonoscopy-related 2	1 (3.3 %)
Patients with ≥ 1 procedure-related SAE	0 (0 %)

AE, adverse event; SAE, serious adverse event.

1 Bowel preparation-related AEs were nausea and vomiting.

2 Colonoscopy-related AE was abdominal pain.

## Discussion

UC is a chronic relapsing and disabling inflammatory disease that exclusively affects the colonic mucosa. Evaluation of the mucosa is essential for diagnostic purposes, but also has prognostic value. Endoscopy plays an important role not only in the diagnosis but also in the management and follow-up of patients with UC. Some evidence indicates that a mere sigmoidoscopy may not be enough to appropriately evaluate disease activity 28 .

The mere absence of symptoms is not a reliable marker for MH, and MH is associated with improved long-term outcomes; therefore, the mucosa should also be evaluated in asymptomatic UC patients and treatment strategy adjusted accordingly 4 8 9 . This current paradigm of management of UC based on a “treat-to-target” approach, undoubtedly leads to a higher number of endoscopic procedures in each single UC patient 15 . Histologic healing is an emerging prognostic tool that requires biopsies of endoscopically healed mucosa for histologic evaluation. The clinical importance of histologic mucosal healing has yet to be defined 29 .

Fecal calprotectin has been shown to be a reliable surrogate marker for disease activity 30 . Despite the widespread use of fecal calprotectin, it may not be enough to avoid all endoscopic procedures in UC patients: the lack of a clear and validated cutoff level to accurately discriminate between patients in deep remission and patients with mucosal inflammation, may lead to the performance of an endoscopic procedure. If fecal calprotectin is high, an endoscopic procedure may be indicated to confirm the presence of disease activity, especially in asymptomatic patients. Although fecal calprotectin is a useful tool, endoscopic procedures will often be needed in UC management to implement the “treat-to-target” paradigm 31 32 .

Colonoscopy can be an unpleasant, embarrassing or even painful procedure. Patient experience is part of quality control of proper disease management 33 . Acceptance of endoscopic procedures plays an important role in obtaining patients’ compliance with current therapeutic objectives. Reliable and comfortable methods beyond surrogate markers of disease activity would be useful. Cost, procedural risk, and endoscopy unit availability may limit the use of colonoscopy in every UC scenario.

Our feasibility study has shown that SBC-CE is practicable, well-tolerated, and reliable in the evaluation of the mucosa in UC patients with a panenteric capsule endoscopy. Our results demonstrate a moderate agreement between the SBC-CE and optical colonoscopy for disease extent (κ = 0.42) and a very good degree of agreement between SBC-CE and conventional colonoscopy findings (κ = 0.86) based on the Mayo score, especially discriminating patients with absence or presence of mucosal inflammation, including Mayo endoscopic sub-score 1. Colonoscopy identified five patients in remission, whereas SBC-CE identified four of these five in remission and one patient to have a Mayo endoscopic sub-score of 1. This is very relevant since MH is the therapeutic objective in the “treat-to-target” approach with definite impact on clinical outcomes.

Grading of inflammatory disease activity is important as well since this too has a prognostic significance and therapeutic implications 14 34 35 36 . Consequently, SBC-CE may become a useful tool for UC management. Disease activity evaluated by SBC-CE showed a high degree of agreement with colonoscopy findings according to the Mayo endoscopic sub-score. Even though there are different endoscopic scoring systems for UC, there is no validated and widely accepted definition for MH.

Nevertheless, Mayo score is commonly used in UC since it is simple, reliable, and useful. A Mayo endoscopic sub-score of 1 is usually considered to indicate MH, and it is also true that some studies indicate that patients with a Mayo score of 0 fare better than patients with a Mayo score of 1 5 9 . A meta-analysis conducted by Shah et al. demonstrated that those UC patients who achieved a more demanding definition of mucosal healing, such as Mayo endoscopic sub-score 0, had better long-term outcomes than patients with a higher score 9 .

The possibility of evaluating the mucosa by capsule endoscopy may represent a new opportunity for many UC patients in different clinical scenarios that are yet to be defined by further research. This may include surveillance of UC patients in remission during the follow-up to confirm mucosal healing, monitoring response to therapy, or to rule out the presence of inflammatory activity of the disease if clinically unclear.

At this point, cancer surveillance for chronic UC patients requires colonic biopsies, and for this reason, SBC-CE is not a viable alternative. Our feasibility study did not address cost effectiveness or patient preference of SBC-CE versus colonoscopy/sigmoidoscopy. A study by D’Haens et al. found a clear patient preference for SBC-CE over colonoscopy in a cohort of Crohn’s disease patients 37 . Further studies will define clinical indication, accessibility, availability, cost effectiveness, patient preference, and safety of SBC-CE in patients with UC.

SBC-CE also provides information on the small-bowel mucosa. In this small study, as much as 3/23 (13 %) cases of previously diagnosed UC had small-bowel involvement as well. Findings in the small bowel may change the diagnosis of UC to indeterminate colitis or to Crohn’s disease. In some cases, the cause of refractory UC may be an incorrect diagnosis and the proportion of patients with this condition may be much higher than would usually be expected. Generalization of panenteric endoscopy in presumably UC patients may provide a new perspective on the nature of the disease itself and probably also some clues with regard to prognosis and refractoriness.

In our study, the degree of agreement between SBC-CE and conventional colonoscopy with regard to disease extent was moderate (κ = 0.42), although we did not analyze the colon segment by segment. When patients with disease limited to the rectum and those with left-sided colitis were grouped together, the agreement of extent of disease between the capsule and colonoscopy improved (κ = 0.61). The clinical relevance of disease extent is usually of lesser importance in clinical management than the presence of mucosal inflammation itself 15 . It must be taken into account that part of the disagreement in the assessment of UC extent may lie in the fact that the accurate distinction in patients with longstanding or active UC may be difficult, even with conventional colonoscopy, especially in those in which haustration is somehow absent and differentiating segments is usually a triggering challenge; moreover, there is no currently available device that measures distance traveled.

For all the above-mentioned reasons, differentiating proctosigmoiditis from proctitis or left-sided colitis seems a very specific and critically demanding objective for a capsule device, and this must be considered in further studies or if elucidating the role that SBC-CE may play in the management of UC. Nevertheless, SBC-CE seemed accurate enough to discriminate between extensive or pancolonic UC and proctitis or left-sided colitis when categorized together; those are forms of UC that may be treated topically, and even though the distinction between proctitis and left-sided colitis may be of some clinical relevance in some cases, we could not address this issue in our study. This may be a limitation of the capsule itself (despite current technological developments) or may be related to the small sample of patients included. Additional improvements in the software or in reader training may help SBC-CE become more accurate in differentiating rectal disease from left-sided colitis; however, further research is needed to properly address the reliability and usefulness of assessing disease extent with an SBC-CE device.

In conclusion, the use of a panenteric capsule endoscopy system seems feasible and reliable in the evaluation of UC. SBC-CE may provide enough relevant information for clinical management of UC, without the disadvantages of conventional colonoscopy. More studies are needed to appropriately define the proper role of this new tool in the clinical management of UC.

## Disclaimer

This study was used for an international market. The PillCam Crohn’s system received CE mark in 2016 and FDA clearance in 2017. The PillCam Crohn’s capsule has not been cleared for use in ulcerative colitis.
